# Phonological Abilities in Persian Speaking Preschool Children with Stuttering and Fluent Peers

**Published:** 2019

**Authors:** Neda TAHMASEBI, Akram AHMADI, Peyman ZAMANI, Mozhgan NOURAFSHAN, Farzaneh SALEHIMANESH

**Affiliations:** 1 Musculoskeletal Rehabilitation Research Center, Ahvaz Jundishapur University of Medical Sciences, Ahvaz, Iran; 2 Department of Speech Therapy, School of Rehabilitation, Babol University of Medical Sciences, Babol, IR Iran.; 3 Hearing Research Center, Ahvaz Jundishapur University of Medical Sciences, Ahvaz, Iran; 4 Ferdowsi University of Mashhad, Mashhad, Iran

**Keywords:** Stuttering, Speech sound production, Percentage consonant correct, Preschool children

## Abstract

**Objectives:**

Speech sound production is poorer in stutterers than normally fluent peers. This study was performed to compare speech sound production abilities in Persian speaking children with developmental stuttering.

**Materials & Methods:**

Overall, 34 children with stuttering and 60 children without stuttering aged from 3 to 6 yr old were enrolled from Ahvaz City, Khuzestan Province, southern Iran in 2016. The phonetic information test was used to assess speech sound production in this study and 30-minute mother-child conversations were utilized for calculation of Percentage Consonant Correct. Phonological abilities of these two groups were compared against each other and a correlation between stuttering severity and speech sound articulation was calculated.

**Results:**

There was significant difference between children with stuttering and normal peers for articulation error total percentage but not significant difference was found for percentage consonant correct (*P*=0.16). Moreover, no significant correlation between stuttering severity and speech sound production in this population was found.

**Conclusion:**

No association seems to exist between stuttering severity and speech sound production abilities in this population. This study may lead to the notion that there was significant difference between the two groups in speech sound production assessment.

## Introduction

As a subtype of communication disorders (1), developmental stuttering is one of the speech fluency disorders manifested clinically by interruption in speech flow such as repetition, block, and prolongation (2). The prevalence of stuttering is estimated at less than 1% in the adult population (3) and 2.52% in preschool children in the US (4). Stuttering is a multifactorial disorder affected by different factors. Moreover, phonetic and phonological factors attracted attention to themselves in stuttering (5).

Postma and Kolk discussed theory on the production and phonological planning, which contributes to stuttering (6). This theory is consistent with some studies that report the rate of incidence of phonological disorders in children with stuttering is higher than normal peers (7, 8). Children's phonetic and phonological development is very fast between the age of 2 to 4 and this range is considered as stuttering onset in the literature (9-11). Other communication disorders may comorbid with stuttering (12, 13). Concomitant disorders complicate the communication process and cause children with stuttering to feel that communicating is difficult and exhausting. 

A study was conducted on the comorbidity of other communication disorders in children with developmental stuttering. 16.8 of children with developmental stuttering, had articulation disorders too (12). The likelihood of phonological disorders is far more in children with stuttering than fluent peers (14-18), in contrast, there was no significant difference between children with stuttering and normal peers in articulation and phonological abilities (17, 19). Studies in this field can be categorized into two groups. Early studies frequently performed by informal assessment tools, which reported that speech sound production in children with stuttering is poorer than normally fluent peers. More recent studies carried out by formal and standardized assessment tools not always demonstrated a significant difference between children with stuttering and normal peers (20).

Due to different results on speech sound abilities in children with stuttering and normally fluent peers and lack of such study for Persian children with stuttering, we decided to investigate and compare both the percentage of the correct consonant and speech sound production abilities in Persian preschoolers.

## Materials & Methods

**Participants**

Participants were 34 children with stuttering aged between 3-6 yr from Ahvaz City, Khuzestan Province, southern Iran in 2016. Inclusion criteria were Persian monolingual children for both groups, normal oro-motor skills as evaluated by a Persian protocol (21), normal language development assessed by Test of Language Development-Persian3 (TOLD-P3) (22), normal hearing assessed by hearing screening, normal intellectual and cognitive skills confirmed by teachers and parents reports, and perception of speech and language pathologist. Parents' reports were based on the Persian version of Ages and Stages Questionnaire (ASQ) to screen normally developing children. This questionnaire has 5 sub-scales: None of the children in both groups received intervention of speech and language pathology services in the past 6 months. Stuttering diagnosis was made based on diagnosis of two experienced speech and language pathologists who have worked on fluency disorders in children.

**Procedure**

Each child was tested individually. Examiners were two speech and language pathologists who had experience in working with children with stuttering, and were familiar with International Phonetic Alphabet (IPA) and could easily transcribe children's utterances. First, two speech and language pathologists transcribed the children's speech samples independently then they calculated error percentage for each of them, finally, Intraclass correlation coefficient (ICC) was calculated between the scoring of two examiners.

**Speech Sound Production Assessment**

Speech sound production was assessed by a Phonetic Information Test. This test is a colorful picture naming tool with 69 pictures in A4 size to elicit all Persian consonants in initial, medial and final positions. Ghasisin et al. (23) investigated the psychometric properties of this test. They reported high values for content validity and internal consistency for their instrument. The available assessment tool did not explain about scoring for errors; therefore, we had to calculate error percentage for each child. The examiner asked children to name each picture. If a child could not name the picture, semantic and phonological cues were given to him. If the participant were still not able to name the picture, the examiner would ask him to repeat the target word. The examiner would simultaneously transcribe the children’s utterances and then calculated the percentage for each speech sound error types of each child separately. Omission, substitution and distortion are speech sound errors that frequently discussed in literature. 

**Percentage Consonant Correct**

Thirty minutes of connected speech was recorded to calculate Percentage consonant correct (PCC) for each child (24). All of the speech samples were gathered in a quiet room by a microphone (micromic c520 with amplifier Henyh 802). Video camera (Sony HX200v, High-resolution camera) was used to record the video of the connected speech of the children. 

**Calculation of Stuttering Severity**

All children with stuttering were video recorded for this assessment. The Persian version of Stuttering Severity Instrument 4^th^ edition (SSI-4) was used to measure stuttering severity in children of the stuttering group. The reliability of this assessment tool was investigated by some studies in Iran (25, 26). Tahmasebi et al. (27) developed the first version of Persian SSI-4 in Iran. Thirty minutes of connected speech recorded for calculation of PCC was used to assess the stuttering severity. Then Computerized Stuttering Severity Score CSSS-2 was used for calculation of the percentage of stuttered syllables and the frequency was obtained by SSI4. SSI-4 has three subscales including frequency, duration and concomitant behaviors. Frequency was obtained through stuttered syllable percentage in connected speech. Duration was calculated by the average of three of the longest stuttered utterances. Concomitant behaviors were categorized in four classes and scoring of each class ranged from 0 to 5 and was calculated by re-watching recorded video films of participants by the examiner. Finally, the severity score was obtained by summing of the three subscales’ scores. Inter-rater reliability was computed for speech samples randomly selected from all of speech samples. Two examiners transcribed and scored the sample of 10 children.

**Statistics**

SPSS ver. 22 (Chicago, IL, USA) was used for the statistical analysis of the current study. Kolmogorov- Smirnov test was used to investigate the normal distribution of the sample. After the normal distribution of the sample was confirmed, independent sample *t*-test was used to compare mean scores of two groups in speech sound production test and percentage consonant correct. ICC was used to obtain inter-rater reliability between two examiners. Pearson correlation coefficient was used to compute the correlation between stuttering severity and total errors in PIT. The significance level was set at (*P*<0.05).

**Ethical Consideration**

This study was approved by the Ethical Committee of Ahwaz Jundishapur University of Medical Sciences(Code: IR.AJUMS.REC.1394.109). All of the parents filled the informed consent form and all the participants assented to participate in this research. We explained to parents that connected speech of children who stutter will be video recorded. If any child did not cooperate with the examiner, he was eliminated from the study.

## Results

**Descriptive statistics for stuttering in group children with stuttering**

Table 1 is related to the descriptive data of children’s scores in sub-scales of SSI4.

**Comparison between two groups**

*Comparison between groups for speech sound production*

Overall, 94 preschool children (34 children with stuttering and 60 children without stuttering) were allocated in two groups. The findings related to the stuttering group are provided in Table 2.

Regarding the reported sums for different types of speech sound production errors (distortion, omission, substitution and articulation error total) in terms of percentage for each of them, there was no significant difference between the two groups in the distortion percentage of the two groups. However, there was significant difference between the two groups for substitution percentage, omission percentage and articulation error total percentage (Table 2).

*Comparison between groups for PCC*

As shown in Table 3 there was no significant difference in PCC in the two groups. 

**Correlation**

*Correlation between stuttering severity and error articulation*

There was no significant correlation between the mean of errors in speech production and stuttering severity in group of children with stuttering(r=0.28, *P*=0.1). 

*Correlation between Stuttering Severity and PCC scores*

There was no significant correlation between PCC and stuttering severity in group of children with stuttering(r=0.13, *P*=0.46).

*Inter-rater Reliability*

There was a high correlation between two examiners shown by ICC=0.939, Upper band= 0.982, Lower band with % 95 confidence interval.

**Table 1:** Descriptive data for stuttering in group of children with stuttering

**Statistics**					
	Ss.per	Ss.score	Dur.m	Dur.score	Phy.T
N	34	34	34	34	34
Mean± Std. Deviation	5.82±4.82	9.82±3.66	1.58±.955	6.65±1.89	2.94±2.77

**Table 2:** Comparison of errors in speech sound production between children with stuttering and normally fluent peers

	Group	Mean	Std. Deviation	T	*P*-value
D.per	Normal	.277	1.26	-1.64	0.104
	Stutterer	.897	2.41		
O.per	Normal	.303	.997	-2.47	0.015
	Stutterer	1.04	1.88		
S.per	Normal	4.01	5.51	-3.63	0.001
	Stutterer	10.36	11.44		
Aetp	Normal	4.61	6.16	-3.88	0.001
Stutterer	12.29	12.98

D.per: deletion percentage, O.per: omission percentage, S.per: substitution percentage and AETP: Articulation error total percentage

**Table 3:** Comparison between groups for Percentage Consonant Correct (PCC)

	Group	Mean	Std. Deviation	T	*P*-value
PCC	Normal	98.10	3.12	1.38	0.169
Stutterer	96.99	4.61

## Discussion

The aim of the present study was to investigate the comparison of speech sound production errors and PCC in a group of Persian children with stuttering and normally fluent peers and examine whether there is a correlation between stuttering severity and speech sound production error in children with stuttering group. Additionally, the possibility of a correlation between PCC and stuttering severity in Persian children with stuttering was explored.

Furthermore, the current study aimed to compare the speech sound production between children with stuttering and normally fluent peers. This study showed that articulation error total percentage is higher in children with stuttering than normally fluent peers. This finding was in accordance with another study (12, 27). Phonological and articulation disorders are more prevalent in this group than normal peers are. We only compared speech sound production errors in two groups and did not study the prevalence of speech sound disorders in this population. However, our findings are not in line with another study (20). 

There was no significant difference between children with stuttering and normally fluent peers. One reason for this difference may be sample size. Their study was performed on 277 children with stuttering, and this relatively bigger sample size may cause difference in finding. Another reason for difference might be that we did not have an articulation test with standard scores in Persian and had to report percentage of speech sound production errors by the available assessment tool. There was difference between the function of children in Phonetic Information and Percentage Consonant Correct. There was not any significant difference between PCC in two groups but significant difference between two groups in omission, substitution and total percentage of two groups. This controversy is probably related to the nature of difference between these two procedures. Phonetic Information Test measures articulation of all Persian consonants. Frequency of some Persian consonants (like /tʃ/, /ʒ/ and /p/) is low in Persian and these consonants may not be in connected speech samples of children. This difference may be a reason for the controversy of findings. 

The present study revealed that there was no association between stuttering severity and speech sound production in group of children with stuttering. This finding is in line with other studies (20, 28) but not with others (29-30). Overall, 40 children were studied with stuttering aged between 5 to 6 yr old. In children with higher rate of frequency of disfluency, particularly repetition, the rate of articulation disorders was more than without stuttering. Children with stuttering whose stuttering severity was severe had more articulation errors than school-age children with moderate severity of stuttering. Our different finding with some studies may be related to the different speech sound production abilities in this range than school age children. Stuttering is a developmental disorder and speech sound production is influenced by chronological age. Given the different findings in the mentioned studies, factors like gender and age have an effect on speech sound production abilities in persons with stuttering, and therefore, we need to have well-designed studies that consider age, gender, and their interaction on speech sound abilities in groups of children with stuttering. There were some limitations to the present study. When this study was performed, there was not any other valid and reliable test in Persian with different parts for assessment of various dimensions of speech sound production like articulation skills at connected speech level (conversational speech, story retelling, reading orally a passage), so we only used the PIT as a single word test for assessment of speech sound production. Using other procedures for obtaining speech sample gives us invaluable information about phonological abilities in children with stuttering. 

**In conclusion,** we studied only speech sounds production skills and percentage consonant correct. There are some procedures like phonological patterns, rating of intelligibility and stimulability measurement for assessment of phonological abilities. It is recommended to design researches for studying the phonological patterns, intelligibility and stimulability in this population.
